# Psychometric Validation and Nomological Network of the Amsterdam Executive Function Inventory (AEFI) in Ecuadorian Adolescents

**DOI:** 10.3390/bs16101859

**Published:** 2026-10-09

**Authors:** Jose A. Rodas, Nadia Soria-Miranda, Jose E. León-Rojas, Daniel Oleas

**Affiliations:** 1 Escuela de Psicología, Universidad Espíritu Santo, Samborondón 0901952, Ecuador; jrodasp@uees.edu.ec; 2 School of Psychology, University College Dublin, Dublin 4, D04 V1W8 Dublin, Ireland; 3 Facultad de Ciencias Psicológicas, Universidad de Guayaquil, Guayaquil 090510, Ecuador; nadia.soriam@ug.edu.ec; 4 Mind, Brain, and Behavior Research Center, Department of Methodology of Behavioral Sciences, University of Granada, 18071 Granada, Spain; 5 Escuela de Medicina, Universidad de Las Americas, Quito 170124, Ecuador; 6 Dirección de Investigación, Universidad ECOTEC, Samborondón 092302, Ecuador; daoleas@ecotec.edu.ec; 7 Department of Social Anthropology, Basic Psychology and Public Health, Pablo de Olavide University, 41013 Seville, Spain

**Keywords:** executive functions, AEFI, adolescents, cross-cultural validation, item response theory, confirmatory factor analysis, psychometrics, Ecuador

## Abstract

Executive functions (EFs) are vital for cognitive self-regulation and goal pursuit during adolescence, yet validated rating scales remain scarce in Latin America. This study evaluated the psychometric properties, item-level operating characteristics, and nomological network of the Amsterdam Executive Function Inventory (AEFI) in an Ecuadorian adolescent sample (N = 830; aged 12–18 years; M = 14.30, SD = 1.75; 52.77% female). Employing a split-sample derivation and validation approach with ordinal diagonally weighted least squares estimation, the original 13-item structure yielded poor fit. Following item refinement, a revised 10-item, three-factor model across attention, self-control, and planning demonstrated robust cross-validated structural fit (robust CFI = 0.970, robust TLI = 0.958, robust RMSEA = 0.048, SRMR = 0.043) and scalar measurement invariance across early (12–14 years) and late (15–18 years) adolescence. Dimension-specific graded response modelling confirmed adequate item functioning, with measurement precision concentrated across average latent trait levels (θ = −2.0 to 2.0). Nomological network analyses corroborated construct validity via strong associations with self-reported behavioural dysregulation and affective disruption on the SENA and PAI-A, alongside expected cross-informant divergence across family and school reports and limited discriminant separation from somatic complaints. These findings establish the revised 10-item AEFI as a promising, structurally invariant, and psychometrically adequate brief self-report measure for developmental and educational research in Ecuadorian youth.

## 1. Introduction

Executive function (EF) comprises a set of top-down cognitive processes that facilitate goal-directed behaviour, self-regulation, and adaptive functioning across the lifespan (Hendry & Holmboe, 2021; Suwa et al., 2024; Zamora-Lugo et al., 2025). During adolescence, a critical developmental period marked by extensive neurocognitive maturation, intact EF is essential for meeting escalating academic demands, managing affective reactivity, and navigating social relationships (Nyongesa et al., 2019; Thorell et al., 2020). Traditionally, EF has been assessed using performance-based neuropsychological tests (Nyongesa et al., 2019). However, these laboratory tasks have frequently been criticised for their poor ecological validity, as structured testing environments minimise the everyday cognitive and emotional demands under which self-regulation typically falters (Buchanan, 2016; Karr, 2023). Consequently, behavioural rating scales and questionnaires have gained widespread use in clinical and research settings to capture the behavioural expression of executive difficulties in everyday contexts (Gioia et al., 2000; Karr & Fagle, 2026; Roth et al., 2013). Empirical evidence indicates that self-reported executive complaints often correlate more robustly with internalising symptoms, distress, and clinical psychopathology than with objective performance-based indices (Buchanan, 2016; Hlutkowsky et al., 2026; Karr, 2023; Tal et al., 2025).

Despite their clinical utility, many existing behavioural rating scales present practical limitations. Numerous established inventories, such as the Behaviour Rating Inventory of Executive Function (BRIEF; Gioia et al., 2000; Roth et al., 2013), are lengthy, proprietary, and heavily focused on extreme clinical symptoms, making them less suitable for epidemiological research or large-scale screening in non-clinical cohorts (Barrios-Fernández et al., 2021; Karr, 2023; López et al., 2022). To address these constraints, van der Elst et al. (2012) developed the Amsterdam Executive Function Inventory (AEFI). The AEFI is a brief, freely accessible, 13-item self-report questionnaire designed to evaluate three core components of daily-life executive behaviour: Attention, Self-Control and Self-Monitoring, and Planning and Initiative. Standardised initially in a population-based sample of 6730 Dutch adolescents aged 15 to 18 years, the AEFI demonstrated adequate construct validity and internal consistency, offering a practical alternative for assessing everyday executive dysfunction (van der Elst et al., 2012).

With the expanding worldwide use of EF rating scales, establishing the cross-cultural validity of these instruments is an essential psychometric requirement (Jukes et al., 2024; Kusi-Mensah et al., 2022; Obradović et al., 2026). Direct and questionnaire-based assessments of EF frequently presuppose that the underlying constructs operate universally (Jukes et al., 2024). Nevertheless, sociocultural factors, contextual expectations, and educational norms significantly shape both the expression and perception of executive behaviours (Jukes et al., 2024; O’Donald & Calia, 2025). Recent systematic reviews underscore that while many language- and culture-adapted EF rating scales retain acceptable internal consistency and broadly preserve original factor structures, substantial gaps in methodological rigour persist across the literature (Kantor et al., 2026; Osalo et al., 2025). Specifically, modern psychometric techniques—such as item-level analysis, measurement invariance testing, and fine-grained evaluations of item discrimination—remain underreported (Kantor et al., 2026; Neves-Teles et al., 2026). Moreover, there is an acute shortage of psychometrically evaluated EF measures validated for children and adolescents in low- and middle-income countries, where cultural and educational contexts differ markedly from Western industrialised settings (Kusi-Mensah et al., 2022).

The AEFI has shown promise across diverse international contexts, including an adaptation among Spanish university students that confirmed its three-factor structure, reliability, and factorial invariance across sexes and academic disciplines (Escolano-Pérez et al., 2022). Similarly, related short executive inventories have demonstrated satisfactory validity across multiple languages and populations (Fan et al., 2026; Fogel et al., 2024; López et al., 2022). However, the psychometric properties and structural integrity of the AEFI have not yet been evaluated in Latin American adolescent populations. Adolescents in Ecuador represent a unique cultural and socio-educational context, necessitating an empirical examination of whether the AEFI’s theoretical structure and item characteristics function appropriately in this setting.

Furthermore, delineating the nomological network of the AEFI is vital to verify construct validity. Because self-report ratings reflect subjective perceptions of cognitive and behavioural control, they typically exhibit substantial associations with clinical psychopathology and emotional dysregulation (Buchanan, 2016; Karr, 2023; Suwa et al., 2024). Evaluating these associations against multi-informant assessment systems (such as adolescent self-reports alongside teacher and parent ratings) allows for a stringent appraisal of convergent and discriminant validity while accounting for well-documented informant discrepancies (Hlutkowsky et al., 2026; Obradović et al., 2026).

Accordingly, the present study aims to evaluate the psychometric properties of the AEFI in a community sample of Ecuadorian adolescents. The specific objectives are: (1) to evaluate the internal structure of the AEFI using confirmatory factor analysis (CFA); (2) to assess the item-level operating characteristics, discrimination, and scale precision across the latent trait continuum using three separate dimension-specific Graded Response Models (GRMs) within an item response theory (IRT) framework; and (3) to examine the scale’s nomological network through convergent and discriminant validity correlations with established multi-informant and self-report measures of emotional, behavioural, and clinical problems, including the Personality Assessment Inventory–Adolescent (PAI-A) and the Sistema de Evaluación de Niños y Adolescentes (SENA).

## 2. Materials and Methods

### 2.1. Participants

The data for this study derived from a larger project investigating the psychometric properties of psychological assessment tools in an Ecuadorian sample. The final sample for the current study comprised 830 adolescents with a mean age of 14.30 years (SD = 1.75). In terms of gender distribution, 52.77% were female and 47.23% were male. Participants were recruited across three geographical regions of Ecuador: Costa (62.05%), Sierra (27.23%), and Amazonía (10.72%).

### 2.2. Instruments

Executive Functioning Assessment Questionnaire (AEFI). Self-reported executive functioning was assessed using the AEFI (van der Elst et al., 2012). The instrument consists of 13 items designed to capture everyday cognitive and behavioural manifestations of executive control in children and adolescents. Items are rated on a 3-point Likert scale (1 = not true, 2 = partially true, 3 = true). The AEFI comprises three theoretical subscales: attention (evaluating concentration and sustained focus), self-control (measuring impulsivity, motor activity, and inhibitory control), and planning (assessing future-oriented behaviour, task prioritisation, and goal pursuit). Positively worded items within the planning dimension are reverse-scored so that higher scores systematically denote greater perceived executive dysfunction across all domains.

System for Evaluation of Children and Adolescents (SENA): Emotional and behavioural problems, contextual vulnerabilities, and psychological resources were assessed using the SENA (Fernández-Pinto et al., 2015). The instrument adopts a multi-informant assessment paradigm, and the present study utilised the self-report booklet for secondary students (aged 12 to 18 years) alongside the parallel informant-report versions completed by families and teachers. All items are scored on a 5-point Likert scale ranging from 1 (never or almost never) to 5 (always or almost always). To examine the convergent and discriminant nomological validity of the AEFI, specific problem scales were selected across informants: attention problems, hyperactivity–impulsivity, emotional regulation problems, anger control problems, and somatic complaints. Additionally, the learning problems scale was included from the school report, and the composite executive function index was evaluated across all three informant perspectives.

Personality Assessment Inventory–Adolescent (PAI-A): Clinical psychopathology and personality patterns were assessed using the PAI-A (Morey, 2007). The PAI-A is an omnibus, self-administered clinical instrument developed for adolescents aged 12 to 18 years, comprising 270 items scored on a 4-point Likert scale (0 = false, not at all true, 1 = slightly true, 2 = mainly true, 3 = very true). For the nomological network analysis, manifest scores from theoretically relevant clinical scales and subscales were selected: cognitive anxiety (evaluating intrusive ruminations and worry-related concentration disruption), cognitive depression (assessing perceived cognitive inefficiency, helplessness, and negative self-evaluation), borderline features (measuring affective instability, identity disturbance, and impulsive tendencies), antisocial features (capturing egocentricity and rule-violating conduct), and somatic concerns (evaluating health preoccupations and somatic symptoms).

### 2.3. Procedure

The study adhered to the ethical principles of the American Psychological Association and Ecuadorian regulations for research in educational settings. Ethical approval was granted by the Universidad de Guayaquil (Resolution No. R-CSU-UG-SE21-111-20-05-2022). Linguistic adaptation of the AEFI followed standard back-translation procedures. Two experienced clinical psychologists, with cognitive and neuropsychological backgrounds, whose native language is Spanish and who are fluent in English (including one of the authors) independently carried out the forward translation from English into Spanish and subsequent back-translation into English. Semantic, conceptual, and cultural equivalence of the items was reviewed, and minor discrepancies in wording were resolved by mutual consensus to ensure developmental and contextual suitability for Ecuadorian adolescents.

Sampling and institutional access across continental Ecuador were coordinated through formal agreements tailored to each region. In the Coast and Amazon regions, authorization was obtained through a network of fiscomisional educational institutions overseeing schools in those areas, whereas access in the Andean Highlands was coordinated via a non-formal youth educational movement with wide institutional coverage. Data collection occurred in groups of 10 to 25 adolescents using paper-and-pencil formats, coordinated directly with psychology staff from institutional Student Counselling Departments and supervised by a lead psychologist and two support assessors. Informational sessions were conducted with families—in person, or virtually for Highland participants—to outline study objectives and secure parental consent alongside participant assent, with no financial compensation provided.

Prior to analysis, cases were screened for eligibility and completeness; adolescents who did not provide informed assent, lacked parental consent, or failed to complete questionnaires in full were excluded. Consequently, the final self-report AEFI sample comprised 830 adolescents with complete data across all indicators. For the external multi-informant analyses, missingness arose solely from unreturned observer protocols, resulting in effective sample sizes of 830 self-reports, 774 family reports, and 682 school reports, which were evaluated using pairwise deletion.

### 2.4. Data Analysis

To mitigate sample-specific optimisation, the dataset was randomly split into independent derivation and validation subsamples. The internal structure of the AEFI was evaluated using confirmatory factor analysis (CFA). Given the three-category response format, the diagonally weighted least squares (WLSMV) estimator was utilised, as it is specifically designed for ordered categorical data. Goodness of fit was evaluated using the comparative fit index (CFI), Tucker–Lewis index (TLI), root mean square error of approximation (RMSEA), and standardised root mean square residual (SRMR). Measurement invariance across early and late adolescent cohorts was subsequently tested using the WLSMV estimator, with strong (scalar) invariance supported if changes in practical fit indices remained within recommended thresholds (ΔCFI ≤ 0.010, ΔRMSEA ≤ 0.015).

Item functioning was evaluated within an item response theory (IRT) framework. To align theoretically and statistically with the multidimensional factor structure, three dimension-specific unidimensional Graded Response Models (GRMs) were estimated. Model-data fit for individual items was assessed using the S-X2 index. Category characteristic curves and expected item score curves were examined to inspect category threshold functioning and item discrimination. Scale-level precision was determined using the test information function (TIF) and conditional standard error of measurement (CSEM).

The nomological network was assessed using Pearson product-moment correlation coefficients between the AEFI dimensions and external scales from the SENA and PAI-A. Positively worded items comprising the planning dimension were reverse-coded prior to all structural, item-level, and correlational analyses to ensure uniform construct directionality, where higher scores consistently reflect greater executive dysfunction. Significance levels were adjusted using the Holm procedure to control for the family-wise error rate. The internal consistency of the latent dimensions was estimated using ordinal McDonald’s omega (ω) derived from the structural model parameters.

All analyses were performed in the R 4.6.1 statistical computing environment (R Core Team, 2026), utilising the packages dplyr (Wickham et al., 2026) for data preparation, psych (Revelle, 2026) for descriptive and correlational analyses, lavaan (Rosseel, 2012) for confirmatory factor analysis, semTools (Jorgensen et al., 2026) for model-based composite reliability estimation, and mirt (Chalmers, 2012) for item response theory modelling.

## 3. Results

### 3.1. Confirmatory Factor Analysis and Internal Consistency

To mitigate sample-specific optimisation, the full dataset (N = 830) was randomly partitioned into a derivation subsample (n = 415) and an independent holdout validation subsample (n = 415). The internal structure of the AEFI was evaluated using CFA with the WLSMV estimator to account for the three-category ordinal response format. Prior to estimation, the positively worded items comprising the planning dimension were reverse-coded to ensure all latent factors systematically represented executive dysfunction.

In the derivation subsample, the original 13-item, three-factor structure yielded an inadequate fit to the data, χ^2^(62) = 209.55, *p* < 0.001, Robust CFI = 0.815, Robust TLI = 0.767, Robust RMSEA = 0.084, 90% CI [0.068, 0.099], SRMR = 0.085. Inspection of the parameter estimates and modification indices in the derivation subsample revealed substantial structural misspecifications. Specifically, Item 13 demonstrated a negligible primary loading on Planning (ꞵ = 0.14) alongside extensive cross-loadings, most notably on Attention (MI = 83.07) and Self-Control (MI = 81.25). Items 5 and 7 likewise showed poor parameter performance within the Self-Control dimension, characterised by weak standardised loadings (ꞵ = 0.33 and ꞵ = 0.34, respectively) and residual covariances that distorted the latent structure. Consequently, Items 5, 7, and 13 were removed. Although Item 6 also exhibited a modest primary loading on Attention (ꞵ = 0.32), it was retained to preserve construct breadth and ensure sufficient empirical identification for subsequent item-level modelling.

The revised 10-item model was subsequently evaluated in the independent validation subsample. This model demonstrated an excellent structural fit across all evaluated indices, χ^2^(32) = 47.63, *p* = 0.037, Robust CFI = 0.970, Robust TLI = 0.958, Robust RMSEA = 0.048, 90% CI [0.014, 0.075], SRMR = 0.043. As detailed in Table 1, all retained items loaded significantly onto their designated latent factors (*p* < 0.001), displaying acceptable explained variance. Because the scoring direction was harmonised prior to estimation, latent covariances indicated significant positive associations between attention and self-control (r = 0.85, *p* < 0.001), attention and planning (r = 0.72, *p* < 0.001), and self-control and planning (r = 0.42, *p* < 0.001).

Internal consistency of the latent dimensions was evaluated in the validation subsample using ordinal McDonald’s omega (ω) derived from the structural parameters. The Self-Control factor demonstrated acceptable composite reliability (ω = 0.732). Conversely, the Attention (ω = 0.533) and Planning (ω = 0.575) subscales yielded lower reliability estimates.

### 3.2. Item Response Theory Analysis

To align with the multidimensional structure confirmed by the CFA, item operating characteristics were evaluated using three separate unidimensional GRMs for the attention, self-control, and planning dimensions. Model-data fit for individual items was assessed using the S-X2 index. The majority of items demonstrated adequate fit (*p* > 0.05). Although item 6 (attention) and item 8 (planning) yielded statistically significant misfit (*p* = 0.030 and *p* = 0.012, respectively), their associated RMSEA values were ≤0.049, indicating that deviations from the model were negligible.

**Figure 1:**
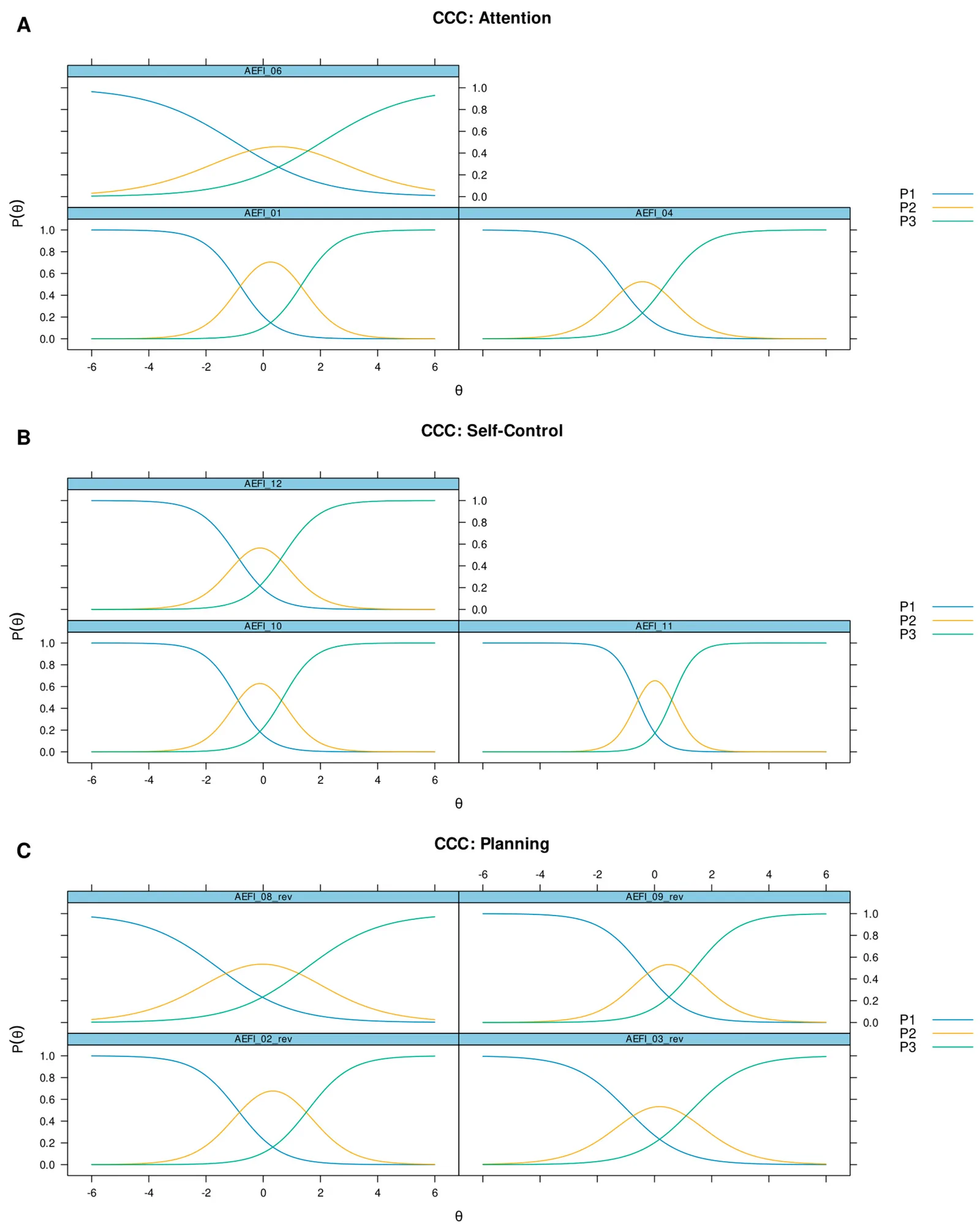
Category Characteristic Curves for the AEFI Items. The horizontal axis represents the latent trait (θ), and the vertical axis represents the probability of endorsing a specific response category (P_1, P_2, P_3). Panel (**A**) represents Attention, Panel (**B**) represents Self-Control, and Panel (**C**) represents Planning.

Visual inspection of the category characteristic curves (see Figure 1) and discrimination parameters (a) indicated strong psychometric properties for most indicators. The self-control items (10, 11, and 12) exhibited excellent discriminative capacity, featuring steep slopes and parameters ranging from 1.56 to 2.52. Items 1 and 4 (attention) and items 2, 3, and 9 (planning) similarly displayed high discrimination (see Table 2). Conversely, items 6 and 8 exhibited lower discrimination, characterised by flatter trajectories across the latent continuum.

**Figure 2:**
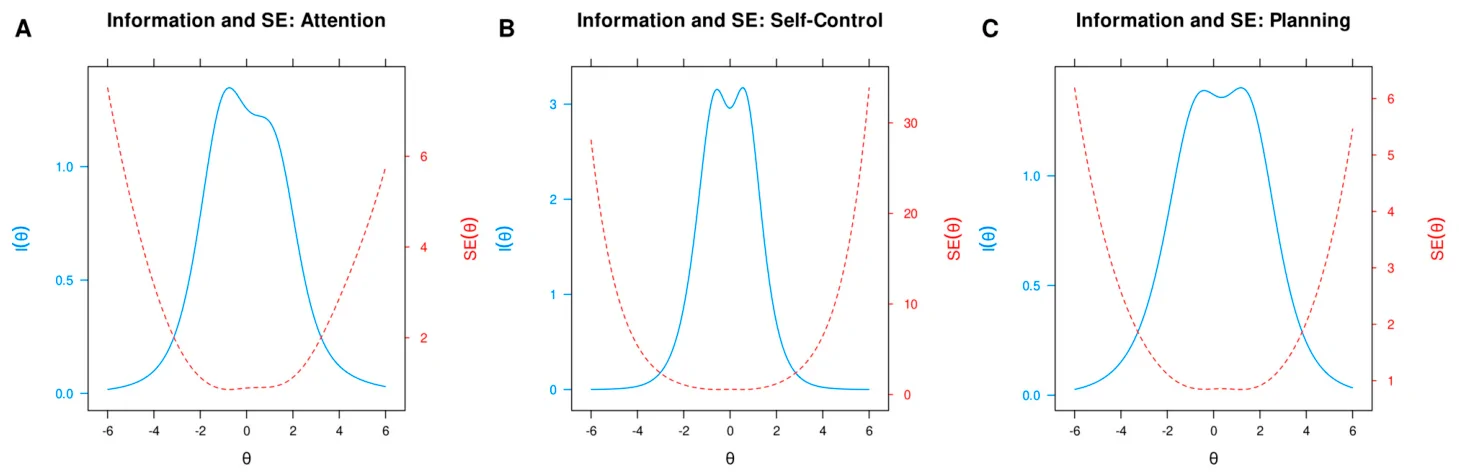
Test Information Functions and Conditional Standard Errors of Measurement for the AEFI Dimensions. The solid blue lines denote the test information function, indicating measurement precision across the latent trait (θ). The dashed red lines denote the conditional standard error of measurement. Panel (**A**) represents Attention, Panel (**B**) represents Self-Control, and Panel (**C**) represents Planning.

At the dimension level, the test information functions and conditional standard errors of measurement demonstrated that the AEFI yields optimal measurement precision for individuals within the average range of executive functioning (see Figure 2). Across all three subscales, measurement information peaked for latent trait levels (θ) between −2.0 and 2.0. Precision declined substantially beyond these bounds, confirming that the instrument operates most effectively for identifying average executive difficulties rather than providing precise measurement at the extreme ends of the trait distributions.

### 3.3. Nomological Network

To establish the convergent and discriminant validity of the revised 10-item AEFI within a broader nomological network, Pearson product-moment correlation coefficients were calculated between the scale scores and a series of theoretically linked external criteria (see Table 3). The relationships were examined across self-report instruments as well as observer-report scales from family and school environments, with significance thresholds adjusted using the Holm procedure to control for family-wise error rate.

#### 3.3.1. Convergent Validity and Self-Reported Clinical Indicators

The revised AEFI demonstrated evidence of same-informant convergent validity through strong associations with self-reported behavioural dysregulation and psychological distress. The AEFI dimensions displayed pronounced positive correlations with core behavioural indicators of executive impairment, most notably between the attention and self-control subscales and SENA attention problems (r = 0.68 and r = 0.55, respectively, *p* < 0.001) as well as hyperactivity–impulsivity (r = 0.68 and r = 0.55, respectively, *p* < 0.001). Conversely, the associations between the AEFI dimensions and the SENA Executive Function Index were comparatively modest (rs ranging from 0.11 to 0.23, *p* < 0.05). This divergence reflects differences in construct operationalisation: while the AEFI focuses primarily on daily behavioural lapses in attentional focus, impulsivity, and organisation, the SENA Executive Function composite captures higher-order metacognitive regulation, emotional management, and academic adaptation.

Regarding clinical psychopathology, elevated executive dysfunction on the AEFI was strongly associated with cognitive manifestations of affective disruption, including cognitive depression (rs ranging from 0.37 to 0.46, *p* < 0.001) and cognitive anxiety (e.g., r = 0.44 for attention and r = 0.42 for self-control, *p* < 0.001) on the PAI-A. Features characterised by behavioural and emotional dysregulation, including borderline features (e.g., r = 0.49 for attention, *p* < 0.001) and anger control problems (e.g., r = 0.38 for attention, *p* < 0.001), also displayed consistent moderate correlations across the dimensions.

#### 3.3.2. Cross-Informant Divergence

A pronounced asymmetry emerged between self-reported parameters and external informant reports, representing an informative but unresolved feature of the validity evidence. While the AEFI dimensions demonstrated large effect sizes when evaluated against other adolescent self-report scales, associations attenuated substantially against observer ratings. Correlations with family-reported parameters were small, reaching statistical significance primarily for the attention and planning dimensions concerning emotional regulation problems (r = 0.15 and r = 0.18, respectively, *p* < 0.001), general executive functions (r = 0.16 and r = 0.15, respectively, *p* < 0.001), and attention problems (r = 0.15 and r = 0.16, respectively, *p* < 0.001). Associations with teacher reports were negligible across all evaluated domains (rs ≤ 0.10, *p* > 0.05 under Holm adjustment). This pattern indicates that evidence for construct convergence is substantially stronger within the same informant than across independent informants, underscoring that self-report ratings reflect subjective internal cognitive friction that cannot be entirely disentangled from shared-method variance.

#### 3.3.3. Discriminant Validity and Somatic Complaints

Discriminant validity was assessed against somatic concerns on the PAI-A and somatic complaints on the SENA—constructs selected as theoretically distinct from executive control. Rather than demonstrating clean separation, the AEFI dimensions correlated moderately with self-reported somatic concerns on the PAI-A (e.g., r = 0.44 for self-control, *p* < 0.001) and somatic complaints on the SENA (e.g., r = 0.45 for self-control and r = 0.44 for attention, *p* < 0.001). This substantial overlap indicates limited discriminant separation within the self-report modality, likely inflated by shared-method variance and general negative affectivity. Although these associations were statistically non-significant in school reports (rs ≤ 0.10, *p* > 0.05 under Holm adjustment), the self-report overlap demonstrates that the current instrument does not achieve clear discriminant isolation from general subjective distress when assessed via self-report alone.

### 3.4. Measurement Invariance by Age

Measurement invariance testing was conducted across early (12–14 years, n = 455) and late (15–18 years, n = 375) adolescent cohorts using the WLSMV estimator. The baseline configural model demonstrated adequate fit to the data, robust CFI = 0.944, robust TLI = 0.921, robust RMSEA = 0.061, SRMR = 0.049. A scalar invariance model, which constrained factor loadings and item thresholds to equality across age groups, also provided an acceptable fit, robust CFI = 0.952, robust TLI = 0.939, robust RMSEA = 0.064, SRMR = 0.052. Although the scaled chi-square difference test was statistically significant, Δχ^2^(7) = 14.51, *p* = 0.043, the changes in practical fit indices were well within the recommended thresholds for establishing invariance (ΔCFI = 0.008; ΔRMSEA = 0.003). These results confirm strong scalar measurement invariance, indicating that the 10-item AEFI evaluates the underlying executive function constructs equivalently across both developmental stages. This structural equivalence supports the psychometric utility of the instrument for younger adolescents alongside the older demographic targeted in the original standardisation.

## 4. Discussion

The present study evaluated the psychometric properties, item-level operating characteristics, and nomological network of the AEFI in a sample of Ecuadorian adolescents. Although behavioural rating scales of executive function are widely used, rigorous cross-cultural validations within Latin American populations remain scarce (Kusi-Mensah et al., 2022). Our findings demonstrate that a revised, 10-item version of the AEFI provides a structurally robust and valid measure, albeit with limited internal consistency in specific domains of self-reported executive functioning, particularly suited for general population research.

Utilising a split-sample derivation and validation approach with an ordinal estimator (WLSMV), the evaluation of the internal structure revealed that the original 13-item, three-factor model (van der Elst et al., 2012) did not adequately fit the data. An inspection of the parameter estimates highlighted significant structural redundancies and suboptimal performance for items 5, 7, and 13. Importantly, the revised 10-item model demonstrated strong scalar measurement invariance across early and late adolescent cohorts, confirming that the instrument functions equivalently regardless of developmental stage. Item 13 exhibited a negligible standardised factor loading and severe cross-loadings, indicating that it did not meaningfully capture the planning construct within this demographic. The removal of these three items resulted in a parsimonious 10-item model that demonstrated excellent fit across all standard indices. This structural refinement suggests that specific behavioural indicators of executive control may not translate equivalently across sociocultural and educational contexts (Jukes et al., 2024; Kantor et al., 2026). The revised model retains the core theoretical dimensions of attention, self-control, and planning while eliminating psychometric noise, yielding a more precise assessment tool. The comparatively limited reliability of the Attention and Planning subscales must be acknowledged when interpreting these specific dimensions.

The application of dimension-specific item response theory models provided critical insights into the scale’s measurement precision across the latent trait continuum, expanding upon previous classical test theory evaluations of the AEFI (Fan et al., 2026). The test information function and conditional standard error of measurement indicated that the AEFI operates with maximum precision for individuals within the average range of executive functioning. Measurement reliability diminishes substantially at the extreme ends of the trait distribution. Consequently, the AEFI operates effectively as a brief self-report measure to assess general executive friction, but it lacks the necessary sensitivity for precise clinical diagnostics among adolescents with severe impairments. At the item level, while the majority of the retained items displayed excellent discriminative capacity, item 8 exhibited a flatter category characteristic curve, indicating lower utility in differentiating between close trait levels.

The nomological network analyses provided strong evidence for the convergent validity of the AEFI. The general and dimension-specific scores exhibited robust positive correlations with self-reported core symptoms of executive impairment, most notably attention problems and hyperactivity–impulsivity. Furthermore, elevated executive dysfunction was moderately to strongly associated with cognitive depression, cognitive anxiety, and borderline features. This convergence confirms that the AEFI successfully captures the subjective burden of executive dysfunction, which shares substantial variance with affective dysregulation and behavioural impulsivity during adolescence (Buchanan, 2016; Karr, 2023).

A prominent finding within the network analyses was the pronounced cross-informant divergence. While the AEFI correlated strongly with other self-report measures, its associations with family-reported parameters were weak, and correlations with school reports were virtually non-existent. This pattern represents an informative but unresolved feature of the validity evidence rather than a definitive confirmation of the nomological network. While cross-informant disagreement is common in psychological assessment (Hlutkowsky et al., 2026; Obradović et al., 2026), the evidence for construct convergence in this study is considerably stronger within the same informant and method than across independent informants. Consequently, the relatively strong self-report associations cannot be entirely separated from shared-method variance. The scale’s reduced sensitivity to severe impairment, as indicated by the item response theory analyses, may partly explain the attenuated correlations with parent and teacher reports, but the substantial divergence indicates that broader cross-contextual validity remains unverified.

Regarding discriminant validity, the AEFI general score demonstrated moderate positive correlations with somatic complaints across self-report forms. Although executive function and somatisation represent theoretically distinct constructs, this overlap likely reflects a broader factor of negative affectivity (Tal et al., 2025). Adolescents experiencing higher executive friction in their daily routines often face chronic academic and social stress, which can manifest concurrently as somatic symptoms. Although this association was statistically non-significant in the school reports (r = 0.08), the substantial overlap within the self-report modality indicates limited discriminant separation, which is likely inflated by shared-method variance and general negative affectivity.

The study has several limitations. First, the reliance on self-report measures introduces significant shared-method variance, which likely inflated the magnitude of the correlations between the AEFI and the clinical criterion scales, rendering the lack of cross-informant convergence an unresolved validity feature (Buchanan, 2016). Second, the study did not include performance-based neuropsychological tasks; consequently, the concurrent validity of the AEFI against objective cognitive indices remains unverified within this population (Nyongesa et al., 2019). Finally, the cross-sectional design precludes inferences regarding the developmental trajectory, temporal stability, or predictive validity of the scale scores over the course of adolescence.

## 5. Conclusions

The findings of this study provide empirical support for a revised, 10-item version of the AEFI as a psychometrically sound, linguistically adapted instrument for evaluating self-reported executive functioning in Ecuadorian adolescents. By eliminating structurally redundant items with poor psychometric performance, the revised model established a robust three-factor structure representing attention, self-control, and planning, while item response theory analyses confirmed optimal measurement precision within the average range of executive functioning. Furthermore, the observed nomological network supported the scale’s construct validity within the self-report modality, demonstrating strong same-informant convergence with clinical psychopathology, though future research incorporating objective neuropsychological indices is required to resolve the observed cross-informant divergence. Overall, the revised AEFI offers a brief, cost-effective, and promising self-report measure for educational and general population research, facilitating the evaluation of everyday executive difficulties in Ecuadorian adolescents.

## Figures and Tables

**Table 1:** Standardised Factor Loadings and Explained Variance for the Revised AEFI Model.

Factor and Item	β	R^2^
Attention		
Item 1	0.59	0.34
Item 4	0.73	0.53
Item 6	0.41	0.16
Self-Control		
Item 10	0.78	0.60
Item 11	0.74	0.55
Item 12	0.75	0.57
Planning		
Item 2 (rev)	0.68	0.46
Item 3 (rev)	0.64	0.41
Item 8 (rev)	0.40	0.16
Item 9 (rev)	0.54	0.30

Note. β = standardised factor loading; R^2^ = proportion of explained variance. All parameter estimates are significant at *p* < 0.001.

**Table 2:** Item Discrimination and Threshold Parameters for the Revised 10-Item AEFI.

Dimension and Item	a	b_1_	b_2_
Attention			
Item 1	1.60	−0.84	1.35
Item 4	1.43	−1.24	0.39
Item 6	0.66	−0.98	2.05
Self-Control			
Item 10	1.81	−0.94	0.70
Item 11	2.52	−0.60	0.64
Item 12	1.56	−0.94	0.71
Planning			
Item 2 (rev)	1.34	−0.89	1.56
Item 3 (rev)	1.07	−0.94	1.30
Item 8 (rev)	0.78	−1.56	1.50
Item 9 (rev)	1.31	−0.40	1.41

Note. a = discrimination parameter; b_1_, b_2_ = category threshold parameters.

**Table 3:** Correlations Between Revised 10-Item AEFI Scores and Criterion Variables.

Measure and Construct	Attention	Self-Control	Planning
PAI-A (Self-Report)			
Anxiety: Cognitive	0.44 ***	0.42 ***	0.21 ***
Depression: Cognitive	0.42 ***	0.37 ***	0.46 ***
Borderline Features	0.49 ***	0.43 ***	0.28 ***
Antisocial Features	0.38 ***	0.34 ***	0.14 ***
Somatic Concerns	0.38 ***	0.44 ***	0.20 ***
SENA (Self-Report)			
Attention Problems	0.68 ***	0.55 ***	0.34 ***
Hyperactivity–Impulsivity	0.68 ***	0.55 ***	0.35 ***
Emotional Regulation Problems	0.08	0.07	0.04
Anger Control Problems	0.38 ***	0.36 ***	0.21 ***
Executive Function Index	0.23 ***	0.20 ***	0.11 *
Somatic Complaints	0.44 ***	0.45 ***	0.28 ***
SENA (School Report)			
Attention Problems	0.04	0.04	0.02
Hyperactivity–Impulsivity	−0.02	0.04	−0.08
Emotional Regulation Problems	0.05	0.03	0.07
Anger Control Problems	0.05	0.05	0.02
Executive Function Index	0.06	0.04	0.08
Learning Problems	0.05	0.01	0.05
Somatic Complaints	0.06	0.10	0.02
SENA (Family Report)			
Attention Problems	0.15 ***	0.07	0.16 ***
Hyperactivity–Impulsivity	0.13 ***	0.00	0.07
Emotional Regulation Problems	0.15 ***	0.07	0.18 ***
Anger Control Problems	0.11 *	0.07	0.13 ***
Executive Function Index	0.16 ***	0.07	0.15 ***
Somatic Complaints	0.09 *	0.07	0.09 *

Note. PAI-A = Personality Assessment Inventory-Adolescent; SENA = Sistema de Evaluación de Niños y Adolescentes. * *p* < 0.05. *** *p* < 0.001. All *p*-values reflect Holm adjustments for multiple comparisons.

## Data Availability

Derived normative scores and copyrighted testing forms from the SENA and PAI remain the intellectual property of TEA Ediciones and are not publicly shareable.

## Abbreviations

The following abbreviations are used in this manuscript:

ADHD	Attention-Deficit/Hyperactivity Disorder
AEFI	Amsterdam Executive Function Inventory
AIC	Akaike Information Criterion
APA	American Psychological Association
BIC	Bayesian Information Criterion
CCC	Category Characteristic Curve
CFA	Confirmatory Factor Analysis
CFI	Comparative Fit Index
CI	Confidence Interval
CSEM	Conditional Standard Error of Measurement
df	Degrees of Freedom
EFA	Exploratory Factor Analysis
EF	Executive Functions
FIML	Full Information Maximum Likelihood
GRM	Graded Response Model
IRT	Item Response Theory
MAD	Median Absolute Deviation
MLR	Robust Maximum Likelihood
PAI-A	Personality Assessment Inventory–Adolescent
RMSEA	Root Mean Square Error of Approximation
SABIC	Sample-Size Adjusted Bayesian Information Criterion
SD	Standard Deviation
SE	Standard Error
SENA	Sistema de Evaluación de Niños y Adolescentes (System for Evaluation of Children and Adolescents)
SRMR	Standardised Root Mean Square Residual
TIF	Test Information Function
TLI	Tucker–Lewis Index
